# Demographic drivers of gut microbiome diversity

**DOI:** 10.1186/s13568-025-01921-6

**Published:** 2025-08-08

**Authors:** Hannaneh Kabir, Matilda Holtz, Justine Choueiri, Mohammad Reza Kaazempur Mofrad

**Affiliations:** 1 Molecular Cell Biomechanics Lab, Departments of Bioengineering and Mechanical Engineering, University of California Berkeley, Berkeley, CA USA; 2 Harvard Medical School and Massachusetts General Hospital, Boston, MA USA; 3 Molecular Biophysics and Integrative Bioimaging Division, Lawrence Berkeley National Lab, Berkeley, CA USA

**Keywords:** Gut microbiome diversity, Demographic factors, Alpha diversity, Beta diversity, QIIME2 analysis, Microbial community composition

## Abstract

The gut microbiome plays a central role in orchestrating metabolic, immune, and neurological functions essential for human health. While extensive research has explored the effects of diseases and pathological conditions on gut microbiome composition, the influence of demographic factors remains underexplored, limiting our understanding of microbiome variations in disease states. This study addresses this gap by investigating the impact of demographic variables, including age, sex, and geography, on gut microbiome diversity in healthy individuals. Using the American Gut Project’s extensive dataset and the QIIME2 bioinformatics pipeline, we conducted a comprehensive analysis of microbial profiles across diverse demographic groups. Our results revealed significant age-related shifts in microbial richness and composition, and geographic location strongly influenced phylogenetic diversity. In contrast, sex exhibited limited impact on microbial diversity within healthy BMI ranges. These findings highlight the critical role of demographic factors in shaping gut microbiome diversity, providing a foundational framework to better contextualize disease-related microbiome variations and advance personalized healthcare approaches.

## Key points

1. Age and geography shape gut microbiome diversity among healthy individuals.
2. Sex has minimal effect on microbial diversity within healthy BMI ranges.
3. Enhanced personalized interventions may arise from contextual microbiome insights.

## Introduction

The human gut harbors a vast and intricate community of trillions of microorganisms—including bacteria, archaea, viruses, fungi, protozoa, and parasites that collectively make up the gut microbiome (Jaswal et al.^2023^; Matijašić et al. 2020). This diverse ecosystem has co-evolved with humans over millennia, forming a symbiotic relationship that is integral to our physiology (Adetunji et al. 2024). Remarkably, our bodies contain more microbial cells than human cells, and the genetic material of these microbes far exceeds that of our genes (Sender et al. 2016). The gut microbiome plays essential roles in digestion, nutrient absorption, and the regulation of metabolism and immune system function. It also influences the development of the nervous system, thereby affecting both physical and mental health (Haller 2018; Krishnamurthy et al. 2023). Bacterial species within the gut are taxonomically organized from broad phyla down to specific strains. The dominant phyla include *Firmicutes*, encompassing genera like *Lactobacillus*, *Clostridium*, and *Enterococcus*, *Bacteroidota*, and *Actinobacteria*. Bacteria in the gut assist in fermenting indigestible fibers to produce short-chain fatty acids such as acetate, propionate, and butyrate (Yen 2019; Afzaal et al. 2022). The compounds exhibit anti-inflammatory, antineoplastic, and antimicrobial properties. Additionally, they regulate metabolic pathways like gluconeogenesis and lipogenesis, while supporting brain function and maintaining the integrity of blood-tissue barriers (Benameur et al. 2023; Nakkarach et al. 2021).

The gut microbiome is initially shaped at birth, influenced by factors such as the mode of delivery (vaginal birth versus cesarean section), infant feeding methods (breastfeeding versus formula feeding), weaning practices, and the mother’s microbiota (Song et al. 2013; Ho et al. 2018). Though established early in life, it remains unique to each individual and continues to develop, impacting long-term health through interactions with host developmental pathways. Advances in sequencing technologies have revealed the gut microbiome’s immense diversity and its critical role in human health and disease (Yen 2019; Jokela et al. 2024). Host factors, including genetics and age, alongside environmental influences such as diet, physical activity, and geography, play pivotal roles in shaping its composition and diversity (Shin et al. 2016; Ren et al. 2023). For example, dietary patterns like Western diets are associated with increased *Firmicutes* and reduced *Bacteroidota*, while regional differences in microbial communities have been documented across populations in China, rural Africa, and Europe (Shin et al. 2016). Lifestyle factors, including smoking, stress, and exercise, further modulate the microbiome, with athletes often exhibiting greater microbial diversity. However, disruptions to this balance, termed dysbiosis, can arise from dietary choices, antibiotic use, medical interventions, and exposure to environmental pollutants (Afzaal et al. 2022; Shapiro et al. 2022). These disturbances can reduce beneficial bacteria while promoting pathogenic strains, diminishing microbial diversity and compromising health. Despite extensive research, understanding how demographic factors collectively influence gut microbiome diversity remains incomplete. Most studies focus on isolated populations or individual demographic factors, limiting the broader applicability of findings. Inconsistencies in study designs, sample sizes, and analytical methods further hinder comparisons across studies. Large-scale investigations that incorporate multiple demographic factors are needed to better understand their combined impact on the gut microbiome (Hrncir 2022; Beiko et al. 2018).

This study aims to conduct a comprehensive assessment of how various demographic factors such as age, sex, and geography are associated with gut microbiome diversity in healthy individuals. By analyzing a large and diverse dataset from the GM Repository (GMrepo), the study seeks to identify patterns and correlations that may not be apparent in smaller or more homogeneous samples. These insights are critical as they provide a foundation to better understand and interpret the variations in the gut microbiome related to diseases and conditions, thus facilitating a more nuanced approach to personalized healthcare interventions.To achieve these objectives, data from the GMrepo, a comprehensive and publicly accessible database compiling gut microbiome sequencing data from healthy individuals worldwide (Dai et al. 2022), were utilized. The GMrepo provides standardized and high-quality datasets, enabling robust comparative analyses across different demographic groups. Leveraging this extensive resource, the study offers valuable insights into the demographic associations with gut microbiome diversity, contributing to the growing body of knowledge in this field (Dai et al. 2022).

## Methods

### Data acquisition from GMrepo

Gut microbiome data for healthy individuals were acquired from the American Gut Project, the largest crowdsourced citizen science initiative focused on human microbiome research. The project has collected samples from various body sites, including fecal, oral, and skin, from thousands of participants across the globe, making it the largest human microbiome cohort in existence. This dataset not only includes participants from North America but also incorporates data from the British and Australian Gut projects, offering a diverse and global representation. Samples used in this study were specifically selected from healthy individuals, and detailed metadata on participants’ health, lifestyle, and dietary habits were included. The American Gut Project’s rich dataset enables an in-depth analysis of associations between the human microbiome and various factors such as diet (from vegan to near-carnivore), seasonal variations, sleep patterns, and conditions like IBD, diabetes, and autism spectrum disorder (McDonald et al. 2018).

In the current study, participants were further grouped based on age, sex, and geographic location to explore potential demographic influences on their gut microbiome. Participant selection was driven by the need for robust comparative analyses across different demographic categories, prioritizing complete demographic metadata and high-quality sequence data. For age groupings, individuals from the US with a healthy BMI were divided into three age groups: 0–18 years, 19–60 years, and 61–100 years, with each group consisting of 20 individuals (Table 1). In terms of sex groupings, the cohort comprised US individuals aged 19–60 with a healthy BMI, split into two groups of 20 individuals based on male and female categories (Table 1). For the geographic location-based grouping, participants were categorized into three groups of 20 individuals, all with a healthy BMI and aged 19–60 years. This group was mixed in terms of sex and sourced from three distinct regions: the US, UK, and Australia (Table 1). The dataset for each group included 16 S rRNA FASTQ files, providing high-resolution microbial diversity and composition data by targeting the 16 S rRNA gene, a widely used marker for identifying and comparing bacteria (Silva et al. 2024). These demographic groupings allow for the investigation of how age, sex, and geographic location may influence the gut microbiome, enabling a more granular understanding of variations across different populations. By incorporating these sequencing files, the study ensures robust microbial profiling for accurate comparisons across population subgroups.

**Table 1:**
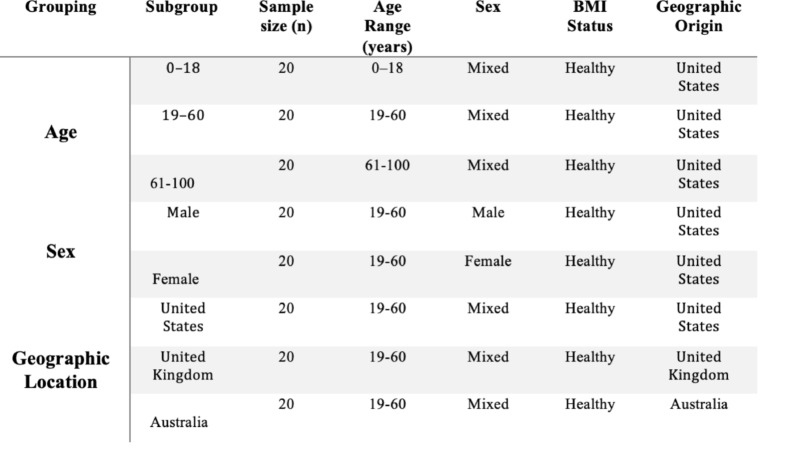
Summary of demographic subgroupings and sample sizes for analysis of gut Microbiome diversity

### Data preprocessing and quality control

In this study, the QIIME 2 pipeline was utilized to preprocess the raw paired-end sequencing data, ensuring the retention of high-quality sequences for downstream analyses. The initial step involved demultiplexing the sequences, as the dataset contained reads from multiple samples within the same file. Since the barcodes had already been removed from the reads and were provided in a separate file, the q2-demux plugin was employed to segregate the sequences according to their respective samples. This step was crucial for organizing the data and eliminating any potential cross-sample contamination.

**Fig. 1:**
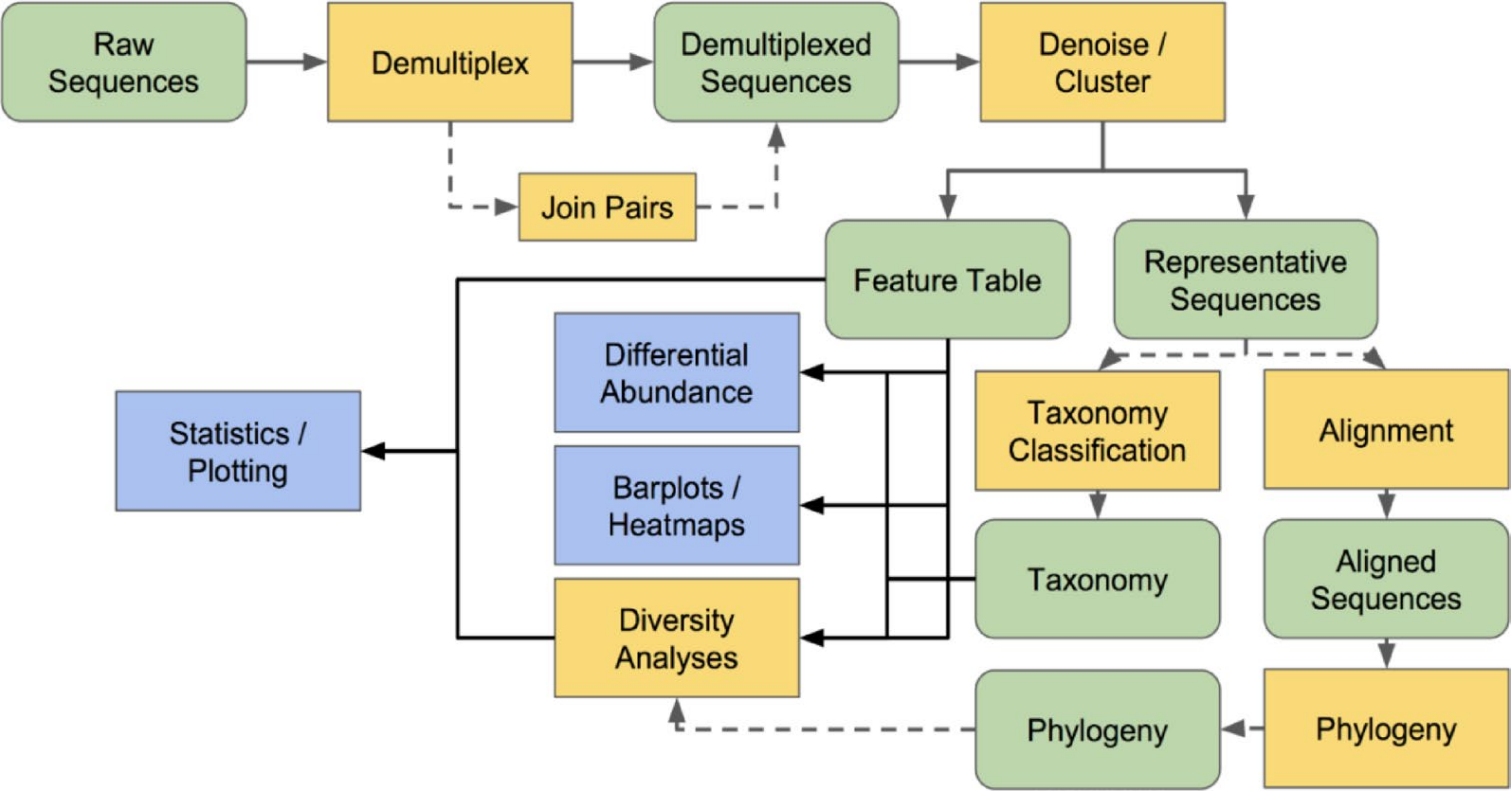
QIIME2 conceptual overview for examining amplicon sequence data

Following demultiplexing, the presence of non-biological sequences such as primers, adapters, and PCR spacers was addressed, as these can interfere with the accuracy of sequence alignment and taxonomic classification. The *q2-cutadapt* plugin within QIIME 2 was used to remove these contaminants from the paired-end data. The *q2-cutadap*t plugin offers comprehensive methods for trimming and filtering non-biological sequences, enhancing the overall quality of the dataset. After cleaning the data, the paired-end reads were merged using the *q2-vsearch* plugin with the merge-pairs method. Merging the forward and reverse reads was essential because the downstream Deblur denoising process requires single-end reads (Fig. 1).

For the denoising step, the Deblur algorithm was employed to correct sequencing errors and remove chimeric sequences. The denoise-16 S method within the Deblur plugin was used, which includes an initial positive filtering step to discard any reads with less than 60% identity to sequences in the 85% similarity GreenGenes database. This stringent filtering reduces the likelihood of including spurious sequences or contaminants. Deblur also performs quality filtering and chimera removal, ensuring that only high-quality, non-chimeric sequences are retained. By integrating these preprocessing steps, demultiplexing, contaminant removal, read merging, and denoising with Deblur, the dataset was prepared to be both accurate and reliable for subsequent microbial community analyses (Fig. 1).

Finally, quality filtering was performed using the quality-filter plugin’s q-score method, which assessed sequences based on their quality scores to remove low-quality reads and enhance the reliability of subsequent analyses. This step ensured that only high-quality sequences were retained for further processing. Following this, sequences were dereplicated using the q2-vsearch plugin’s dereplicate-sequences method. Dereplication consolidated identical sequences, reducing redundancy and computational load in downstream analyses, resulting in a set of unique sequences along with their corresponding frequencies across samples.

### Sequence alignment and feature table construction with QIIME 2

Sequence alignment and feature table construction were performed to accurately represent the microbial communities within the samples. Prior to clustering, essential preprocessing steps were conducted to prepare the data for Operational Taxonomic Unit (OTU) clustering. These steps included merging paired-end reads, removing non-biological sequences such as primers and adapters, trimming reads to a uniform length, and discarding low-quality reads through quality filtering using the quality-filter plugin’s q-score method. This ensured that only high-quality, biologically relevant sequences were retained for analysis (Fig. 1).

Following preprocessing, sequences were dereplicated using the q2-vsearch plugin’s dereplicate-sequences method. Dereplication consolidated identical sequences, reducing redundancy and computational load in downstream analyses, and resulted in a set of unique sequences with their corresponding frequencies across samples. For sequence alignment and feature table construction, closed-reference clustering was implemented using the q2-vsearch plugin’s cluster-features-closed-reference method. This approach grouped sequences matching reference sequences in databases such as GreenGenes or SILVA, which were imported as FeatureData[Sequence] artifacts, at a specified similarity threshold. Closed-reference clustering produced a comprehensive feature table that recorded the frequency of each OTU across samples, laying the foundation for downstream taxonomic and diversity analyses (Fig. 1).

To further investigate microbial diversity and relationships, phylogenetic analyses were performed using the QIIME2 *align-to-tree-mafft-fasttree* pipeline. Initially, sequences were aligned using the MAFFT algorithm implemented within the q2-alignment plugin. MAFFT employs iterative refinement to generate accurate multiple sequence alignments, critical for robust phylogenetic reconstruction. Post-alignment, alignments were masked to remove ambiguous positions or gaps that could negatively impact the phylogenetic inference process. Subsequently, a phylogenetic tree was constructed from these masked alignments using the FastTree algorithm through the q2-phylogeny plugin, which generates approximately maximum-likelihood trees efficiently.

Two types of phylogenetic trees were generated: rooted and unrooted trees. The unrooted tree illustrates relationships among sequences without assuming a common ancestor, providing a basic framework of phylogenetic relationships. The rooted tree was created by applying midpoint rooting to the unrooted tree, a method that identifies the midpoint of the longest path between two tips as the root. Rooted trees facilitate interpretation of evolutionary relationships and enable meaningful downstream diversity analyses, including calculation of phylogenetic diversity metrics like Faith’s Phylogenetic Diversity and UniFrac distances (Fig. 1).

### Taxonomic classification

Taxonomic classification of the microbial sequences was performed using the QIIME 2 feature-classifier plugin, specifically employing the classify-sklearn method. This machine learning-based approach utilizes a Naive Bayes classifier to assign taxonomy to sequences based on patterns learned from reference databases. A pre-trained classifier was downloaded from the QIIME 2 data resources page, which was trained on a comprehensive database such as SILVA or GreenGenes. This method enhances classification accuracy by leveraging extensive reference data and allows for adjustments in classification parameters to refine the results. By using the classify-sklearn method, accurate and efficient taxonomic assignments were achieved.

### Diversity analysis

#### Alpha diversity metrics

To study richness and evenness of microbial communities, facilitating comparisons across different age, sex, and geographic location groups, alpha diversity metrics were calculated using QIIME 2’s q2-diversity plugin. Specifically, Shannon’s diversity index, a quantitative measure of community richness, and Pielou’s evenness were employed to assess the diversity within each sample. The core-metrics-phylogenetic method was applied, which involves rarefying the FeatureTable[Frequency] to a specified sampling depth to ensure even sampling across all samples. This rarefaction minimizes biases due to varying sequencing depths by randomly subsampling counts so that each sample has the same total count. The choice of sampling depth was made by reviewing the total counts in each sample and selecting a value that retains as many sequences as possible while excluding as few samples as necessary. These alpha diversity metrics provided insights into the richness and evenness of microbial communities, facilitating comparisons across different age, sex, and geographic location groups.

#### Beta diversity metrics

To identify potential patterns and dissimilarities in microbial communities among age, sex, and geographic location groups, beta diversity metrics were calculated. Three beta diversity metrics were calculated to assess the differences in microbial community composition between samples. The Jaccard distance, a qualitative measure, was used to evaluate community dissimilarity based on the presence or absence of features (e.g., operational taxonomic units). The Bray-Curtis distance, a quantitative measure, accounted for differences in feature abundances between samples, providing insight into the extent of compositional variation. Additionally, the weighted UniFrac distance was employed, which incorporates both the abundance of features and their phylogenetic relationships to assess community dissimilarity. This metric offers a more comprehensive view by considering evolutionary distances between microbial taxa.

### Statistical analysis of demographic associations

To investigate the relationships between gut microbiome diversity and demographic variables such as age, sex, and geography, a comprehensive suite of statistical approaches was employed. Alpha diversity metrics including Shannon’s diversity index and Pielou’s evenness were analyzed using the non-parametric Kruskal-Wallis test to detect group-level differences. Where significant, Dunn’s post-hoc tests were conducted, with Benjamini-Hochberg corrections (adjusted q-values) applied to control the false discovery rate and yield q-values.

Differences in microbial taxonomic composition across demographic groups were assessed using the ANCOM test within the QIIME 2 platform, which accounts for the compositional nature of microbiome data. Additional Kruskal-Wallis tests were performed at the genus and species levels for selected taxa, with multiple testing corrections again applied. These analyses provided robust statistical validation for patterns observed in taxonomy bar plots.

For multivariate analysis, Redundancy Analysis (RDA) was conducted using the vegan package in R. This constrained ordination method, applied to the centered log-ratio transformed feature table, quantified the proportion of variation in microbial composition explained by age, sex, and geographic location. Significance of explanatory variables was tested using permutation tests (*n* = 999). Additional multivariate tests, including PERMANOVA and pairwise comparisons, were performed to support the observed associations.

### Visualization and data interpretation

Visualization techniques were employed to interpret and present the results, enhancing the understanding of microbial community composition in relation to sample metadata. Principal Coordinates Analysis (PCoA) plots were generated using the Emperor tool to explore patterns within the data. These plots were based on Jaccard, weighted UniFrac and Bray-Curtis distance metrics and included custom axes such as days since the experiment start, which is particularly useful for examining time series data. The PCoA plots displayed principal coordinate axes that facilitated the visualization of similarities and differences in microbial communities among samples over time.

## Results

### Taxonomic classification

#### Sex grouping

**Fig. 2:**
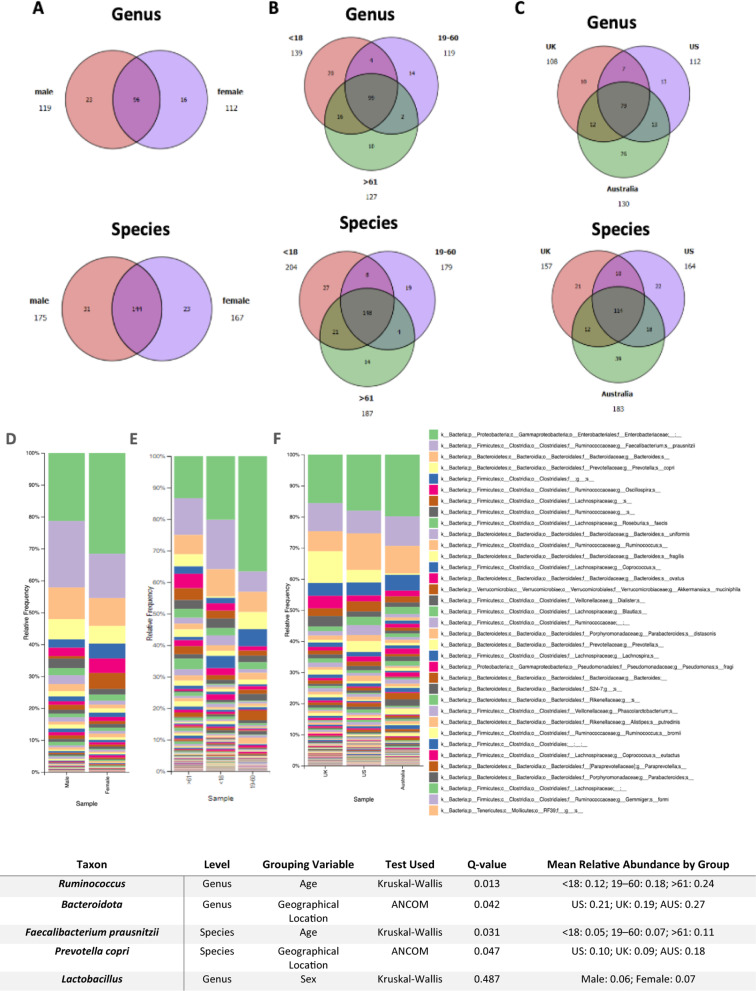
Taxonomic classification: **a** Venn diagrams at genus and species levels for **A** sex, **B** age, and **C** geographic location grouping, and **b** bar plot at species level for **D** sex, **E** age, and **F** geographic location grouping

The taxonomy bar plot in the Sex Grouping analysis compares the relative abundance of bacterial taxa between male and female samples. It highlights sex-specific variations in the gut microbiome. The most abundant phyla, such as *Firmicutes* and *Bacteroidota*, appear prominently in both sexes, but there are notable differences in the proportions of certain bacterial families and genera. For instance, certain taxa within the *Firmicutes* phylum (e.g., *Ruminococcaceae* and *Lachnospiraceae* families) may display a higher relative abundance in one sex compared to the other, reflecting potential differences in diet, hormones, or lifestyle factors (Fig. 2, D). The Venn diagrams complement this by quantifying the shared and unique taxa at the genus and species levels. Specifically, males and females share 96 genera, while males have 119 and females 112, showing a blend of common and unique genera. At the species level, males and females share 144 species, with each sex hosting additional unique species (175 for males and 167 for females). This indicates a substantial core microbiome shared between sexes but with distinct taxa unique to each (Fig. 2, A).

#### Age grouping

In the Age Grouping analysis, the taxonomy bar plots display changes in the relative abundance of bacterial taxa across the three age categories: under 18, 19–60, and over 61. The data suggests dynamic shifts in the gut microbiota composition with age. In younger individuals (< 18), certain bacterial groups, potentially including members of the *Bacteroidota* phylum, may dominate, reflecting dietary habits rich in carbohydrates and simple sugars. In the 19–60 age group, there might be greater microbial diversity with increased abundance of taxa associated with fiber digestion, such as those in the *Ruminococcaceae* family. For individuals over 61, changes in microbial composition may reflect reduced diversity, with an increase in opportunistic taxa potentially linked to aging and associated health conditions (Fig. 2, E). The Venn diagrams reinforce these observations by quantifying the shared and unique microbial taxa across age groups. All groups share a core microbiome of 99 genera and 148 species, but the unique genera (139 for < 18, 119 for 19–60, and 127 for > 61) and species highlight age-specific microbial adaptations. These differences underscore how factors such as diet, physical activity, and health conditions influence microbial community structure over a lifetime (Fig. 2, B).

#### Geographic location grouping

The taxonomy bar plots in the geographic location grouping analysis reveal regional differences in the gut microbiome composition across the UK, US, and Australia. While the dominant phyla (e.g., *Firmicutes and Bacteroidota*) are consistent across regions, specific bacterial families and genera exhibit notable variation. For example, certain genera associated with fiber-rich diets may be more prevalent in Australian samples, whereas taxa linked to high-fat or processed diets might be enriched in the US samples. The UK samples may represent an intermediate microbiome composition influenced by a mixed dietary pattern (Fig. 2, F). The Venn diagrams further illustrate these regional differences by summarizing shared and unique microbial taxa. A total of 79 genera and 114 species are shared across all three regions, reflecting a global core microbiome. However, each country hosts a unique set of additional taxa, likely driven by environmental factors such as diet, healthcare systems, and lifestyle. These regional differences in microbial composition emphasize the influence of geography on gut health and may provide insights into country-specific health outcomes and disease prevalence (Fig. 2, C).

To statistically validate the visual differences observed in taxonomy bar plots, ANCOM and Kruskal-Wallis tests were performed at the genus and species levels. Taxa that showed significant variation across groups (q < 0.05) are reported in the table included in Fig. 2, including their mean relative abundances by demographic group and adjusted *q*-values. These results confirm the presence of age and geographical location-associated microbial shifts, while differences by sex were not statistically significant despite some visual variation.

### Diversity analysis

#### Alpha diversity metrics

##### Sex grouping

**Fig. 3:**
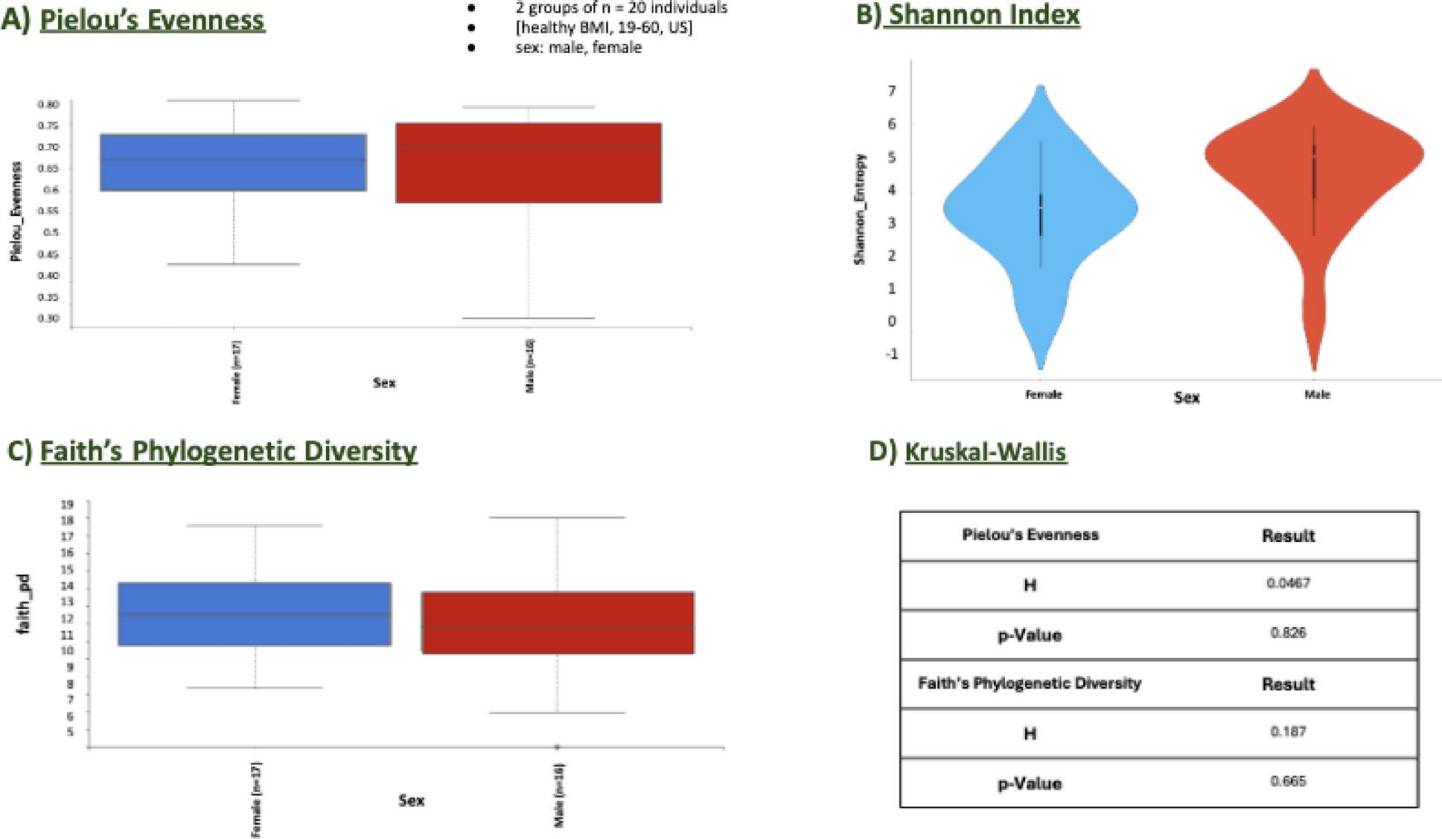
Alpha Diversity Metrics for the sex grouping **A** Pielou’s Evenness, **B** Shannon Index, **C** Faith’s Phylogenetic Diversity, and **D** Kruskal-Wallis

The alpha diversity in sex groupings was analyzed using measures of phylogenetic diversity and evenness across male and female groups within the US dataset. Alpha diversity was assessed through metrics such as Faith’s Phylogenetic Diversity and Shannon Index (Fig. 3B, C). For the US group, the Kruskal-Wallis test for Faith’s Phylogenetic Diversity produced a non-significant result (*p* = 0.665), indicating no statistically significant difference in diversity between males and females (Fig. 3, D). Similarly, the Pielou’s Evenness and Shannon Index for this group showed no significant variation between the sexes, with p-values well above the standard significance threshold (0.826 for Evenness and 0.983 for Shannon Index) (Fig. 3, A). These consistent findings across both tests indicate that, within this sample and under healthy BMI conditions, sex does not significantly impact the observed alpha diversity in microbial communities (Fig. 3).

##### Age grouping

**Fig. 4:**
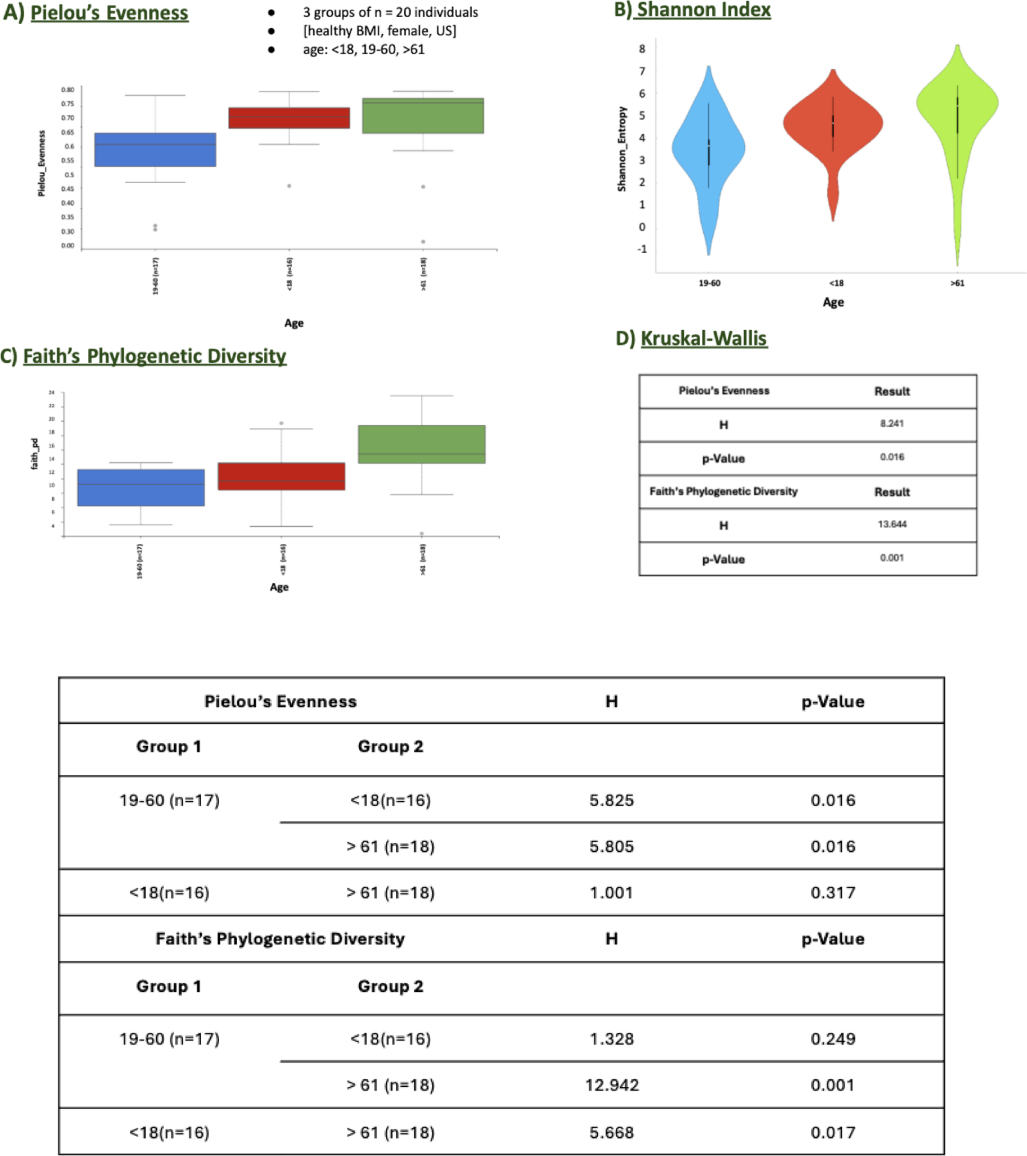
Alpha Diversity Metrics for the age grouping **A**) Pielou’s Evenness, **B** Shannon Index, **C** Faith’s Phylogenetic Diversity, and **D** Kruskal-Wallis

In Age Grouping, which included 20 female participants with a healthy BMI from the US, three age categories were used: <18, 19–60, and > 61 years. Significant differences were observed across these age groups in measures of microbial diversity, as evidenced by both Faith’s Phylogenetic Diversity (Fig. 4, C) and Pielou’s Evenness (Fig. 4, A). The Kruskal-Wallis test (Fig. 4, D) indicated statistically significant variation in Faith’s Phylogenetic Diversity (H = 13.564, *p* = 0.001). Pairwise comparisons revealed that the > 61 age group displayed significantly higher diversity compared to the 19–60 age group (H = 12.942, *p* = 0.001) and the < 18 group (H = 5.668, *p* = 0.017). These findings suggest that microbial community richness increases with age, particularly in individuals older than 61 years.

For Pielou’s Evenness, the Kruskal-Wallis test also showed statistically significant differences (H = 8.281, *p* = 0.016). Pairwise comparisons highlighted differences between the 19–60 group and the < 18 group (H = 5.825, *p* = 0.016), as well as between the 19–60 group and the > 61 group (H = 5.805, *p* = 0.016). However, no significant difference was observed between the < 18 group and the > 61 group (H = 1.001, *p* = 0.317). These results suggest that evenness, or the uniformity of microbial taxa, tends to vary with age, though the differences are less pronounced than those seen in Faith’s Phylogenetic Diversity.

##### Geographic location grouping

**Fig. 5:**
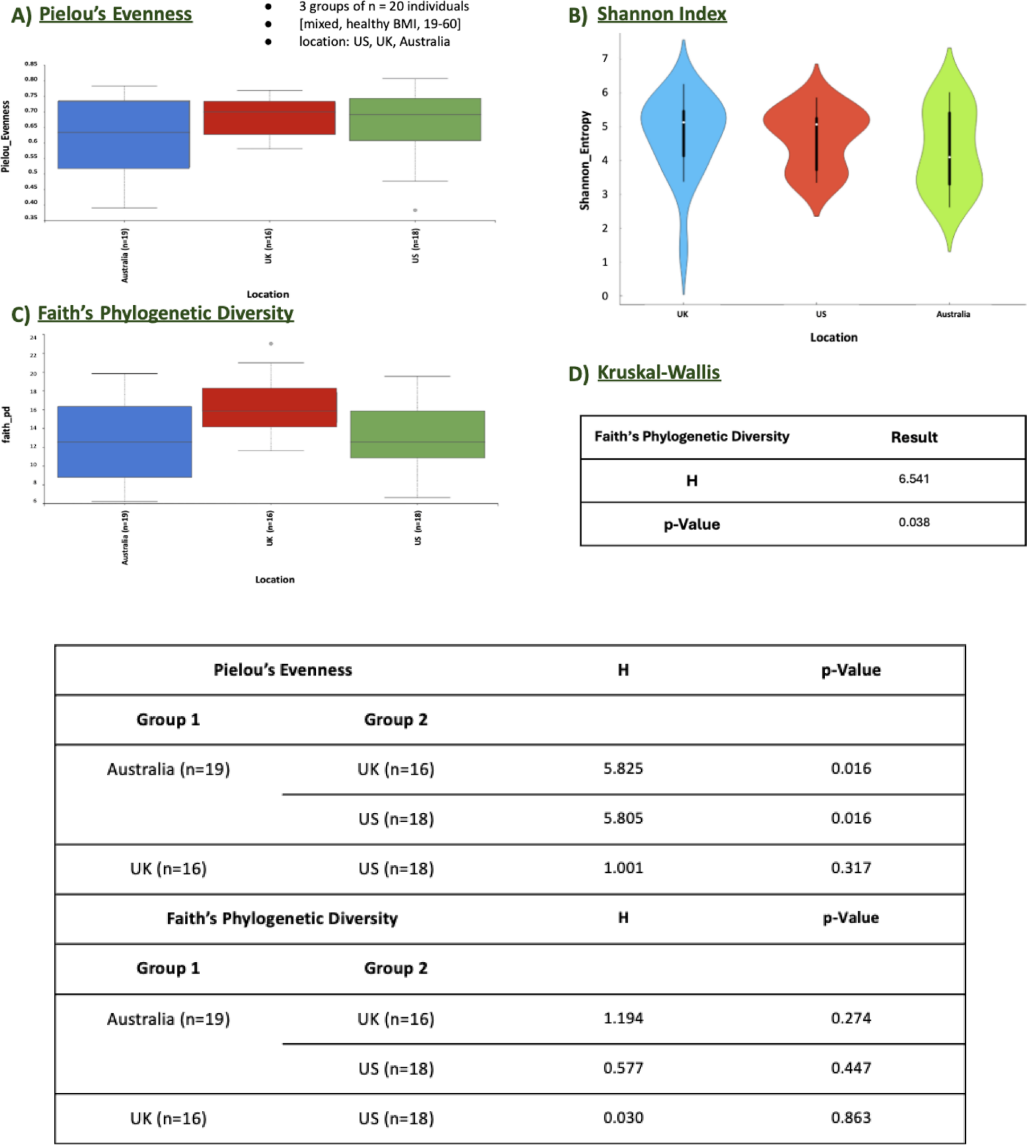
Alpha Diversity Metrics for the geographic location grouping **A** Pielou’s Evenness, **B** Shannon Index, **C** Faith’s Phylogenetic Diversity, and **D** Kruskal-Wallis

The alpha diversity analysis across geographic locations (Australia, UK, and US) provided insights into the microbial diversity among populations from these regions. Pielou’s Evenness and the Shannon Index were used to assess species evenness and richness, respectively. The Kruskal-Wallis test (Fig. 5, D) did not reveal significant differences across geographic locations for Pielou’s Evenness (H = 1.194, *p* = 0.274) (Fig. 5, A) or the Shannon Index (Fig. 5, B), indicating that microbial community evenness and richness were relatively consistent across samples from Australia, the UK, and the US. Pairwise comparisons for Pielou’s Evenness further supported this, with all comparisons showing non-significant p-values (e.g., Australia vs. UK, *p* = 0.016; Australia vs. US, *p* = 0.016), suggesting similar distribution patterns across geographic locations. This was visually confirmed by the violin plots of the Shannon Index, which showed overlapping patterns across the three regions.

Faith’s Phylogenetic Diversity (Fig. 5, C), on the other hand, revealed significant geographic variability. The Kruskal-Wallis test for Faith’s Phylogenetic Diversity showed a statistically significant difference (H = 6.541, *p* = 0.038), suggesting that phylogenetic richness of the microbial communities differed by geographic location. Pairwise comparisons identified significant differences between Australia and the UK (*p* = 0.037) and between the UK and the US (*p* = 0.017), while differences between Australia and the US were not significant. These results indicate that samples from Australia tended to exhibit slightly higher phylogenetic diversity compared to the UK and the US, while the UK showed distinct phylogenetic characteristics compared to the other regions.

In summary, while Pielou’s Evenness and the Shannon Index suggest consistency in microbial richness and evenness across the geographic regions, Faith’s Phylogenetic Diversity points to geographic location-specific variations, particularly between Australia and the UK as well as between the UK and the US. This highlights the potential influence of regional factors on the phylogenetic diversity of microbial communities.

#### Beta diversity metrics

##### Sex grouping

**Fig. 6:**
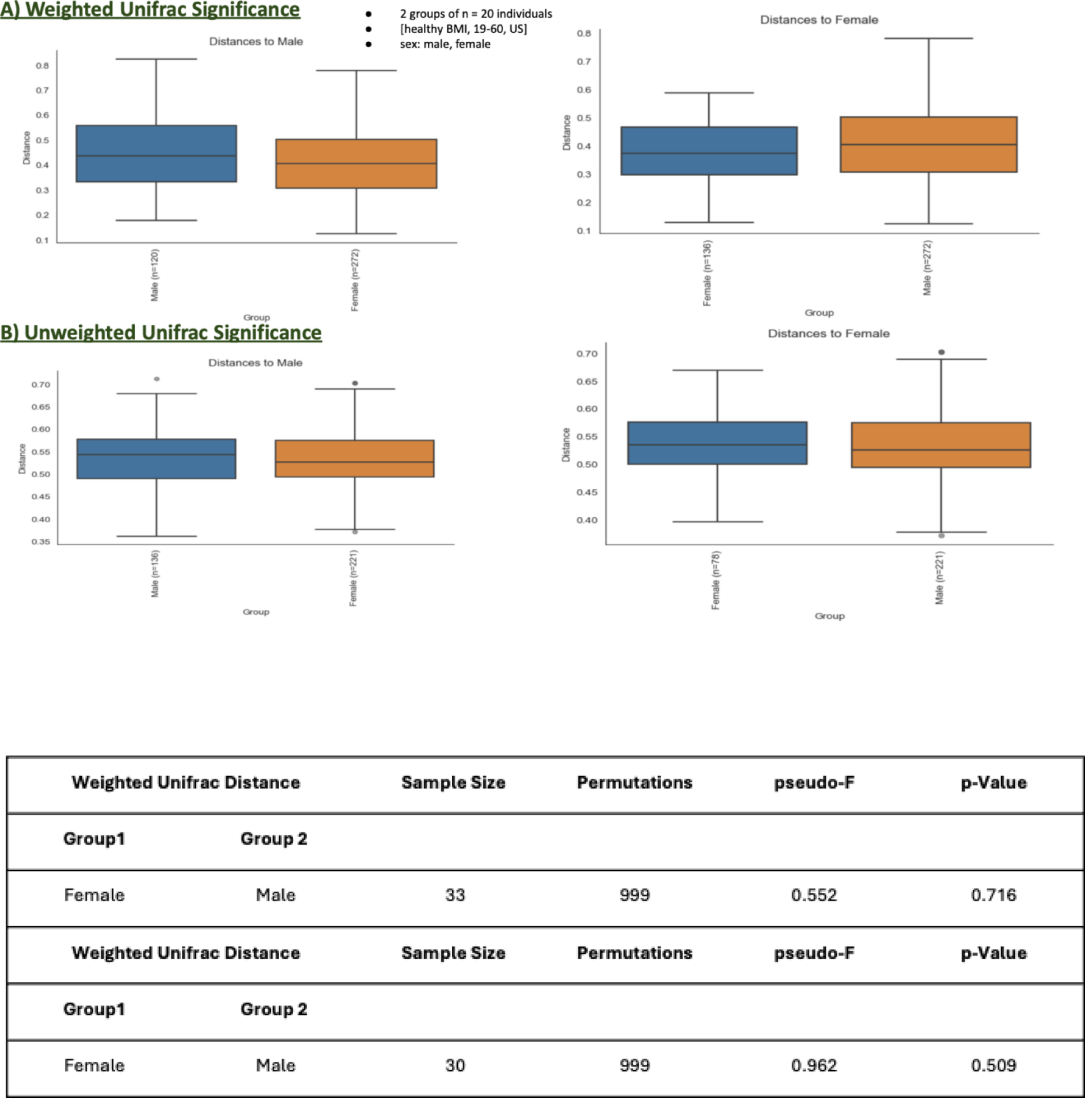
Beta Diversity Metrics for sex grouping **A** weighted unifrac significance and **B** unweighted unifrac significance

The beta diversity analysis based on sex grouping explores differences in microbial community composition between males and females. The analysis was conducted using both weighted and unweighted UniFrac distances to assess microbial community variation. The weighted UniFrac analysis (Fig. 6, A), which accounts for the relative abundance of microbial taxa, showed no statistically significant differences in microbial community composition between males and females. The pseudo-F value was 0.552, and the p-value was 0.716, indicating no meaningful separation between the two groups in terms of microbial abundance. The boxplots reveal similar median distances within and between males and females, further supporting the lack of substantial differences in the abundance-weighted microbial community structure. The unweighted UniFrac analysis (Fig. 6, B), which considers only the presence or absence of microbial taxa, also demonstrated no significant differences between the sexes. The pseudo-F value was 0.962, and the p-value was 0.509, indicating that microbial composition based solely on presence/absence is not significantly different between males and females. Although slight differences in median distances are observed in the boxplots, they do not reach statistical significance. The results from both weighted and unweighted UniFrac analyses suggest limited evidence of sex-based differentiation in microbial community composition. While minor variations in community structure can be observed, these differences are not statistically significant in this dataset.

##### Age grouping

**Fig. 7:**
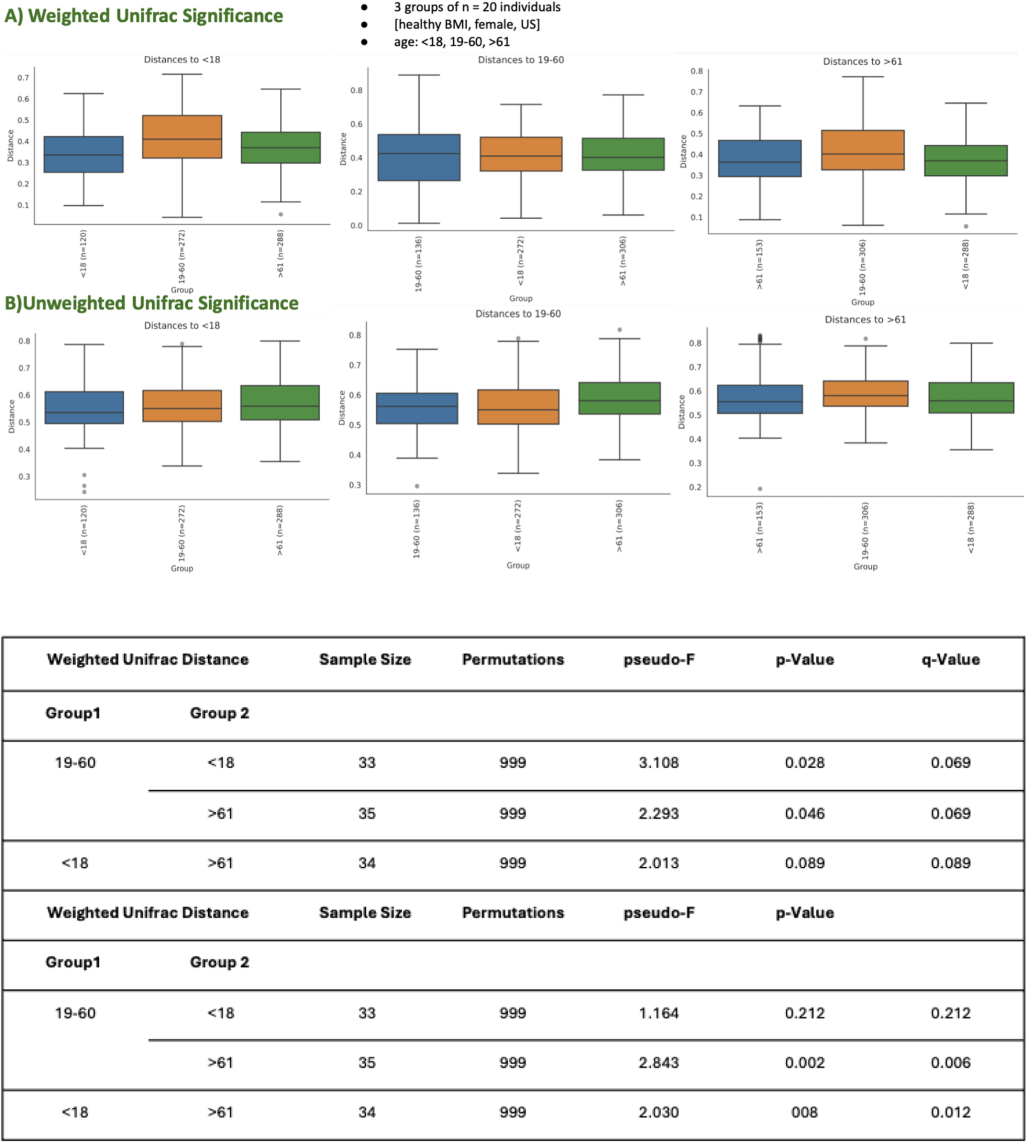
Beta Diversity Metrics for age grouping **A** weighted unifrac significance and **B** unweighted unifrac significance

The beta diversity analysis across different age groups (< 18, 19–60, and > 61) explores microbial community differences across these categories, assessed through both weighted and unweighted UniFrac distances. The findings reveal variations in microbial composition influenced by age. Weighted UniFrac (Fig. 7, A), which accounts for microbial abundance, indicates significant differences between age groups, though the p-values are slightly higher compared to unweighted UniFrac (Fig. 7, B). Specifically, the comparison between the 19–60 and < 18 groups shows a statistically significant difference (pseudo-F = 3.108, *p* = 0.028, q = 0.069), indicating some degree of variability in microbial abundance. Significant differences are also observed between the 19–60 and > 61 groups (pseudo-F = 2.293, *p* = 0.046, q = 0.069), reflecting changes in microbial composition with age. Differences between the < 18 and > 61 groups show a trend toward significance (pseudo-F = 2.013, *p* = 0.089), suggesting greater microbial abundance diversity between these age extremes. Unweighted UniFrac, which considers only the presence or absence of microbial taxa, highlights even more pronounced differences: Significant differences are observed between the 19–60 and > 61 groups (pseudo-F = 2.843, *p* = 0.002, q = 0.006), suggesting distinct microbial compositions between middle-aged and older individuals. The < 18 and > 61 groups also show significant differences (pseudo-F = 2.030, *p* = 0.008, q = 0.012), emphasizing that microbial presence and absence vary considerably between younger and older populations. Comparisons between the < 18 and 19–60 groups do not show significant differences (pseudo-F = 1.164, *p* = 0.212), suggesting more similarity between these two age groups in terms of microbial taxa presence. The beta diversity analysis demonstrates that microbial community composition and abundance vary significantly with age, particularly when comparing the older (> 61) group to the younger (< 18) and middle-aged (19–60) groups. Weighted UniFrac results highlight shifts in the abundance of microbial species with age, while unweighted UniFrac emphasizes differences in the presence or absence of taxa. These findings suggest that age is a key factor influencing the structure of microbial communities, potentially reflecting physiological or environmental changes associated with different life stages.

##### Geographic location grouping

**Fig. 8:**
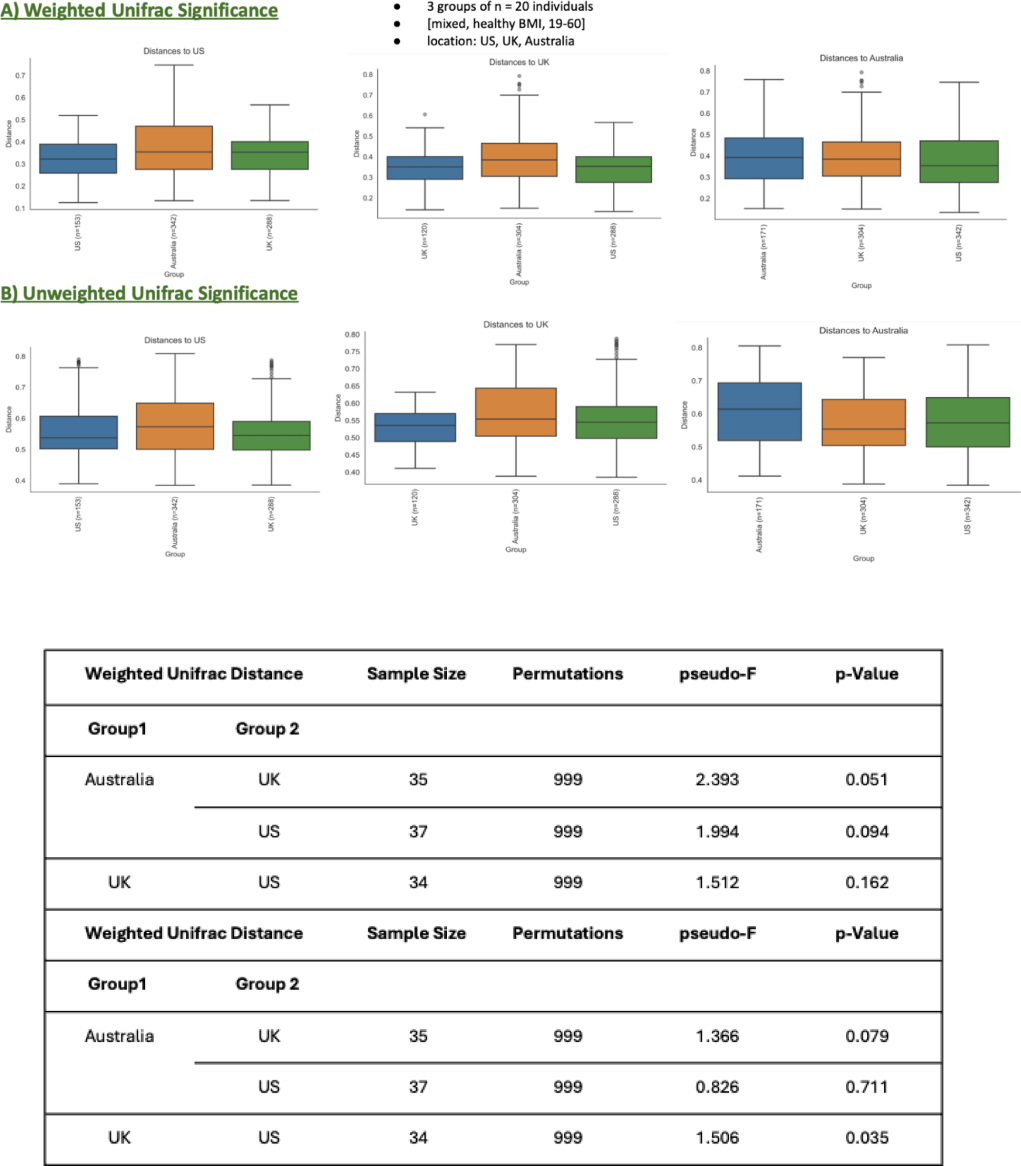
Beta Diversity Metrics for geographic location grouping **A** weighted unifrac significance and **B** unweighted unifrac significance

The beta diversity analysis by geographic location compares microbial community composition across three geographic regions: the US, UK, and Australia. This analysis was conducted using both weighted and unweighted UniFrac distances, providing insights into microbial variation across these populations. The weighted UniFrac analysis (Fig. 8, A), which incorporates the relative abundance of microbial taxa, revealed some trends of variation in microbial diversity between the regions. The comparison between Australia and the UK showed a marginally significant result, with a pseudo-F value of 2.393 and a p-value of 0.051. This indicates a potential difference in microbial abundance between these two regions, though it does not meet the conventional significance threshold. The comparison between Australia and the US yielded a pseudo-F value of 1.994 and a p-value of 0.094, suggesting a weaker but noticeable trend in abundance-related differences. The UK and US comparison had the least pronounced difference, with a pseudo-F value of 1.512 and a p-value of 0.162, reflecting a lack of significant variation in microbial community abundance. The unweighted UniFrac analysis, which evaluates microbial presence or absence rather than abundance, showed more distinct regional differences (Fig. 8, B). Microbial communities differed significantly between UK and US populations (pseudo-F = 1.506, *p* = 0.035). This indicates notable variation in the microbial community composition between these regions when only the presence/absence of taxa is considered. The comparison between Australia and the UK showed a pseudo-F value of 1.366 and a p-value of 0.079, indicating a trend toward significance but not reaching the conventional threshold. The Australia and US comparison had the weakest distinction, with a pseudo-F value of 0.826 and a p-value of 0.711, reflecting minimal differences in microbial presence between these regions. The beta diversity analysis reveals that microbial diversity varies modestly across geographic locations, with more pronounced differences in species composition (as shown by unweighted UniFrac) compared to species abundance (as shown by weighted UniFrac). The significant unweighted UniFrac result between the UK and the US highlights distinct microbial community structures in these populations. However, comparisons involving Australia showed less differentiation, suggesting more similarity in microbial diversity between this region and the others.

To further enhance the interpretability of the PERMANOVA-based beta-diversity analysis, R^2^ values were calculated and reported, quantifying the proportion of variance in microbial community composition explained by each demographic factor. Sex had a minimal impact on gut microbiome composition, with R^2^ values of 0.014 (weighted UniFrac) and 0.025 (unweighted UniFrac), indicating negligible differentiation between males and females. In contrast, age explained a more substantial proportion of variance, with R^2^ values of approximately 0.098 (weighted UniFrac, adults aged 19–60 vs. younger individuals < 18) and 0.091 (unweighted UniFrac, adults aged 19–60 vs. older individuals > 61), suggesting notable differences in microbial communities across age groups. Geographic location also demonstrated a moderate effect on microbial composition, with R^2^ values of 0.078 (weighted UniFrac, Australia vs. UK) and 0.050 (unweighted UniFrac, UK vs. US), underscoring that geography meaningfully contributes to differences in gut microbiome structure. Collectively, these findings highlight the differential influences of demographic factors, with age and geographic location exerting more pronounced effects compared to sex in shaping gut microbial diversity.

### Visualization and data interpretation

The PCoA plots provide a visualization of microbial community composition differences across various demographic groupings, as assessed by the Jaccard distance, which is based on the presence or absence of microbial species. Each plot represents different groups, including sex, age, and geographic location, with ellipses encapsulating the variance for each category within the respective group. These ellipses aid in illustrating how distinct or overlapping the microbial communities are across these demographics.

**Fig. 9:**
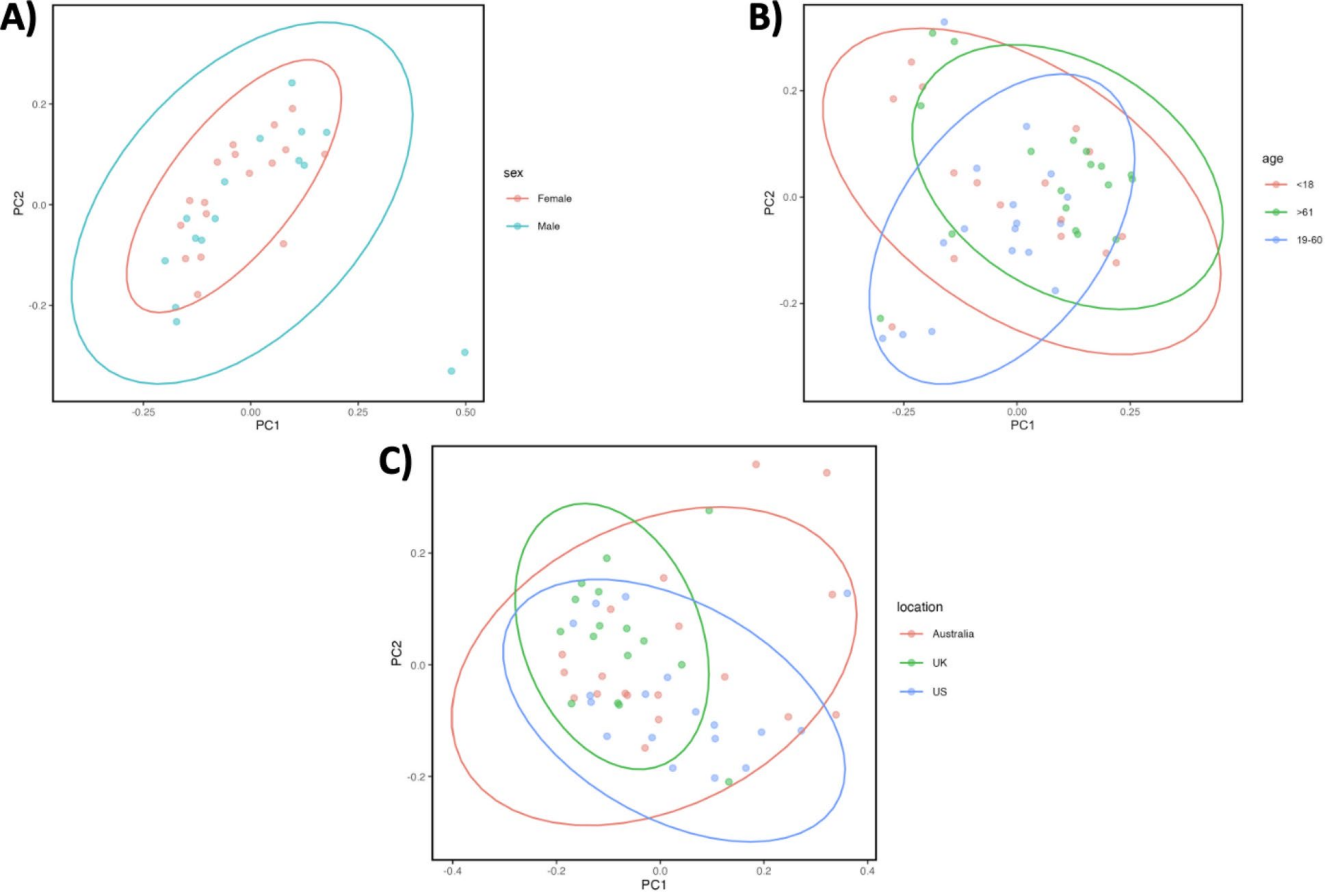
PCoA Plots for the sex, age, and, and geographic location grouping

In the sex grouping plots, the clusters for male and female groups are mostly overlapping with slight separation along the PC1 axis, indicating that there are some compositional differences, but they are not strongly distinct. This suggests that while microbial communities in males and females might differ slightly, these differences are not significant enough to form well-separated clusters. This observation aligns with the previous statistical analyses showing limited significance in microbial community composition based on sex (Fig. 9, A).

The PCoA plot for age groupings shows some clustering with more distinct separation for individuals aged > 61 compared to the younger groups (< 18 and 19–60). While the ellipses exhibit overlap, their orientation indicates a clearer compositional shift in microbial profiles for older individuals. This observation aligns with other analyses suggesting that age-related changes in microbial composition become more pronounced in older adults (Fig. 9, B).

In the geographic location-based PCoA plot, the geographic groups (Australia, UK, and US) display some separation, with ellipses that do not fully overlap, especially between Australia and the other two geographic locations. This plot suggests more distinct microbial composition differences between individuals from different regions compared to sex or age groupings. The US and UK communities have some overlap, while Australia appears more distinct. This supports previous findings of significant geographic location-based differences in microbial diversity, particularly in terms of presence/absence of specific species (Fig. 9, C).

## Discussion and future works

This study enhances microbiome research by providing a focused, controlled analysis of how demographic factors age, sex, and geographic location shape gut microbiome diversity in healthy individuals. Unlike large-scale initiatives such as the American Gut Project, which rely on self-reported data from diverse populations with varying health conditions, this research employs a structured methodology with defined participant groupings and rigorous statistical analyses. By utilizing high-quality data from the GM Repository and the QIIME2 bioinformatics pipeline, confounding factors are minimized, allowing for a more precise understanding of microbial diversity patterns.

The findings reveal significant age-related shifts in microbial richness and composition, as well as geographic variations in phylogenetic diversity, highlighting the critical role of demographic context in shaping gut microbial communities. Redundancy Analysis (RDA) further supported these patterns, showing that age and geographic location explained a substantial proportion of the variance in microbial community composition (adjusted R^2^ = 0.34, *p* < 0.01), while sex contributed minimally (adjusted R^2^ = 0.032, *p* = 0.19). These results reinforce earlier diversity analyses and demonstrate that microbial functional potential varies along key demographic axes. Notably, sex exhibited minimal influence within healthy BMI ranges, emphasizing the multifactorial nature of microbiome dynamics. Together, these insights offer a refined perspective on microbiome variability and provide a strong foundation for future research aimed at personalized healthcare and microbiome-informed interventions (Table 2).

**Table 2:**
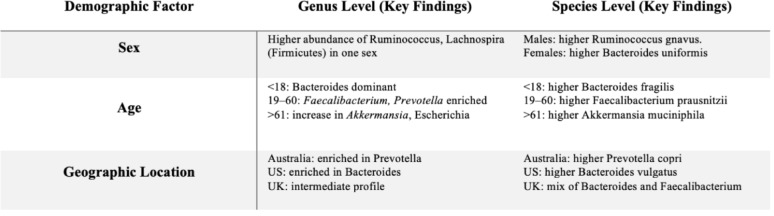
Key microbiome differences across demographic factors

Significant age-related shifts in microbial richness and composition were observed, particularly in individuals over 60 years old. These findings align with previous studies reporting changes in gut microbiome diversity with age. The observed increase in diversity in older adults may be attributed to dietary changes, lifestyle factors, and physiological adjustments associated with aging. For example, Claesson et al. (2012) found that elderly individuals living in community settings exhibited higher microbial diversity compared to those in long-term care, emphasizing the importance of environmental factors in shaping the gut microbiome.

A limited impact of sex on microbial diversity within healthy BMI ranges was noted, contrasting with earlier studies that reported sex-specific differences in gut microbiome composition. However, the findings are consistent with more recent large-scale studies suggesting that sex-related differences in microbiome composition become less pronounced with age. This highlights the complex interplay between age, sex, and other factors in determining gut microbiome diversity and composition (Ira et al. 2024).

Geographic location emerged as a significant factor influencing phylogenetic diversity. This observation is supported by prior research demonstrating that individuals from different geographical regions harbor distinct gut microbial communities. Factors such as diet, lifestyle, and environmental exposures unique to specific geographic locations likely contribute to these geographic variations. For instance, Yatsunenko et al. (2012) reported significant differences in gut microbiome composition between individuals from the United States, Malawi, and Venezuela, attributing these variations to dietary and lifestyle differences. Similarly, Tasnim et al. (2017) found that children from rural communities exhibited more diverse gut microbiotas compared to children from Western populations, further emphasizing the influence of environment and lifestyle on gut microbiome composition.

Future research is encouraged to expand this analysis by incorporating additional demographic variables such as socioeconomic status, dietary patterns, and genetic predispositions to build a more holistic understanding of microbiome diversity. Although this study provides valuable insights, the sample size of 20 individuals per demographic group, while sufficient to identify key trends, could be increased in future studies to enhance statistical power and generalizability. A post hoc power analysis conducted using Faith’s Phylogenetic Diversity as the outcome metric evaluated the adequacy of the current sample size (*n* = 20 per group). This analysis indicated a high effect size (f = 0.878) and statistical power of 1.0 for age-based comparisons, confirming sufficient sensitivity to detect differences. Geographic location comparisons yielded acceptable statistical power (0.724), demonstrating moderate sensitivity to regional effects on phylogenetic diversity. Conversely, sex-based comparisons revealed a very small effect size (f = 0.059) and low statistical power (0.065), aligning with the observed non-significant results. These findings validate the current sample size for detecting substantial demographic effects, particularly for age and geography, while highlighting that limited statistical power may contribute to the absence of significant sex-related differences. Longitudinal studies will be essential to uncover causal relationships and dynamic changes in microbial composition over time, addressing factors not fully accounted for in this study, such as dietary patterns, medication use, and lifestyle habits. Integration of metagenomic, metabolomic, and proteomic data could further elucidate the functional implications of observed microbiome variations. Additionally, the study’s geographic focus on populations from the United States, the United Kingdom, and Australia provides an initial perspective on geographic variations but underscores the need to include underrepresented regions such as Asia, Africa, and South America. Capturing a more complete picture of global microbiome diversity and exploring interactions between the microbiome and host health outcomes across diverse populations will be critical for translating these findings into tailored therapeutic strategies to improve health and disease management.

## Data Availability

Gut microbiome data for healthy individuals from different groups in QIIME2 format is available on Google Drive (request access), https://drive.google.com/drive/folders/14vCITOg7qm3Eccq98MpkzB1GMJfkzmMx? usp=sharing.
